# Influence of Spondylolysis on Clinical Presentations in Patients With Lumbar Degenerative Disease

**DOI:** 10.7759/cureus.12570

**Published:** 2021-01-08

**Authors:** Yasuchika Aoki, Hiroshi Takahashi, Arata Nakajima, Masahiro Inoue, Go Kubota, Takayuki Nakajima, Yusuke Sato, Junya Saito, Koichi Nakagawa, Seiji Ohtori

**Affiliations:** 1 Department of Orthopaedic Surgery, Eastern Chiba Medical Center, Togane, JPN; 2 Department of Orthopaedic Surgery, University of Tsukuba, Tsukuba, JPN; 3 Department of Orthopaedic Surgery, Toho University Sakura Medical Center, Sakura, JPN; 4 Department of Orthopaedic Surgery, Sawara Prefectural Hospital, Katori, JPN; 5 Department of Orthopaedic Surgery, Graduate School of Medicine, Chiba University, Chiba, JPN

**Keywords:** spondylolysis, spondylolisthesis, lumbar spine surgery, lumbar degenerative disease, prognosis, prevalence, computed tomography (ct)

## Abstract

Background: There is insufficient current information regarding the prognosis of patients with lumbar spondylolysis when bone union is not achieved. To examine the number, age, and surgically treated levels of patients with lumbar degenerative disease who underwent lumbar spine surgery, and to compare the results between patients with spondylolysis and without spondylolysis, a cross-sectional study was performed.

Methods: Patients with degenerative lumbar disease who underwent lumbar spine surgery were retrospectively reviewed (n=354). The prevalence of spondylolysis was determined using CT images. Patients were divided into a spondylolysis group and a non-spondylolysis group, and the patients’ age, sex, and surgically treated levels were compared between the two groups.

Results: The prevalence of lumbar spondylolysis in the 354 patients was 6.50% (23/354). The patients’ age was significantly lower in the spondylolysis group (54.2 ± 13.5 years) than in the non-spondylolysis group (63.8 ± 14.2). The number of surgically treated levels was significantly lower in the spondylolysis group (1.33 ± 0.56 levels) than in the non-spondylolysis group (1.70 ± 0.87). The percentage of patients who underwent surgery at L5-S1 was significantly higher in the spondylolysis group; whereas the percentage of patients who underwent surgery at L3-L4 or L4-L5 was significantly higher in the non-spondylolysis group.

Conclusions: Our results suggest that the presence of spondylolysis may not increase the incidence of degenerative lumbar spinal disorders requiring spinal surgery. However, spondylolysis patients frequently have severe degenerative disease at one level caudal to the spondylolysis, and infrequently have multilevel lumbar degenerative disease requiring spinal surgery.

## Introduction

Lumbar spondylolysis, a stress fracture of the pars interarticularis, is one of the common causes of low back pain in adolescent patients [1]. The early diagnosis of spondylolysis can be made by magnetic resonance imaging (MRI) [2,3]. Using MRI, early-stage spondylolysis can be found even when the fracture line is not detected by plain x-ray. Sairyo et al. reported that bone marrow edema in the adjacent pedicle is the indicator for early-stage spondylolysis [3]. In adolescent patients, acute lumbar spondylolysis is usually treated conservatively by the use of a brace and refraining from sports; this, however, often takes long periods of time (three to six months) [4,5]. In some cases, patients resist accepting long-term conservative treatment because they do not want to stop playing sports. Thus, some patients abandon the treatment that could achieve bony union. As a result, the fracture does not heal and it becomes a pseudoarthrosis (pseudoarthrotic spondylolysis) [5]. Understandably, in such cases, the patients (or their parents) may have justified anxiety about their future. At this moment, there is little information about the influence of lumbar spondylolysis on the future occurrence of lumbar degenerative disease in cases where the defect has become a pseudoarthrosis. Because of the lack of information, patients have to make a difficult decision about whether or not they receive long-term conservative treatment.

Beutler et al. studied the natural history of spondylolysis with a 45-year follow-up evaluation. They reported that 19 of 22 patients with bilateral spondylolysis developed spondylolisthesis [6]. Their report is the only prospective study to document the long-term prognosis of spondylolysis over 40 years. Although previous studies demonstrated that spondylolysis patients have higher future incidences of disc degeneration and spondylolisthesis when spondylolysis becomes a pseudoarthrosis [6-11], it is unknown whether the risk for developing severe lumbar degenerative disease requiring surgery increases in these patients.

The purpose of the present study is to clarify how the presence of pseudoarthrotic spondylolysis influences the lumbar degenerative process. First, we examined the prevalence of pseudoarthrotic spondylolysis in lumbar degenerative disease patients who underwent lumbar spinal surgery and compared the results with the general population. If the presence of pseudoarthrotic spondylolysis increases the occurrence of severe lumbar degenerative disease requiring surgical treatment, we hypothesized that the prevalence of pseudoarthrotic spondylolysis in patients who underwent surgery for lumbar degenerative diseases must be higher than that in the general population. Second, we examined the characteristics of lumbar degenerative disease patients who have pseudoarthrotic spondylolysis. We hypothesized that the timing and the level of surgery may differ between patients with spondylolysis and without spondylolysis.

## Materials and methods

Consecutive patients with lumbar degenerative disease who underwent lumbar spine surgery between April 2010 and March 2017 in our institution were included (n=366). Indication for surgery were as follows: intolerable or long-lasting pain (or numbness, on low back, buttock, or lower extremities) that could not be relieved with adequate conservative treatment, progressive or severe neurologic diysfunctions such as muscle weakness, and severe neurogenic bladder dysfunction. Lumbar spondylolysis was not considered when surgical indication was determined.

**Figure 1:**
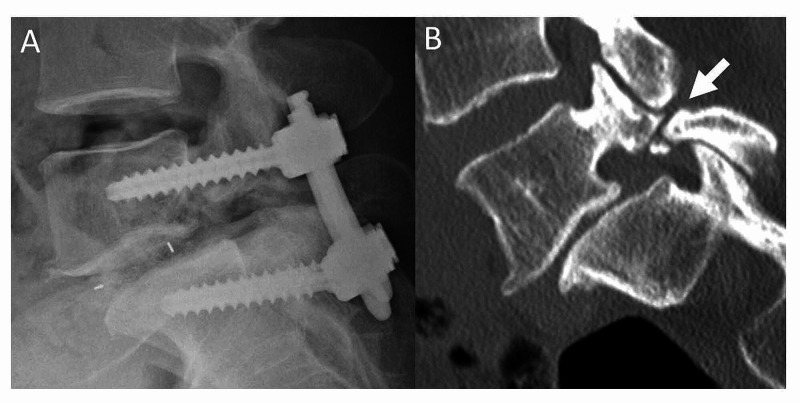
Postoperative radiograph and CT scan A: Post-operative lateral radiograph of a pseudoarthrotic spondylolysis patient who received transforaminal lumbar interbody fusion (TLIF). B: A reconstructed sagittal computed tomography (CT) scan of the same patient taken preoperatively. Arrow indicates a defect of the pars interarticularis. This patient has chronic spondylolysis, which is considered to be pseudoarthrosis.

Patients who had not received preoperative computed tomography (CT) images of the lumbar spine, and those who underwent surgery for pathological conditions other than lumbar degenerative disease (vertebral fracture, infectious diseases, tumors, etc.) were excluded. Finally CT images of 354 patients (96.7%) were included in the present study. Patients’ age, sex, and spinal disease state (disc herniation, spinal stenosis, spondylolisthesis, and others) were reviewed and whether or not they had pseudoarthrotic spondylolysis was determined from the CT images (Figure 1). First, the prevalence of spondylolysis was evaluated. Second, the patients were divided into two groups by whether they have pseudoarthrotic spondylolysis or not (spondylolysis group and non-spondylolysis group). Then, the patients’ age, sex, and surgically treated levels were compared between the two groups. The study protocol was approved by the Institutional Review Board of Eastern Chiba Medical Center (No. 68), and all patients had signed the informed consent.

Values are represented as means ± standard deviation. To compare the prevalence of spondylolysis, the chi-square test was used. To compare the two groups, the chi-square test was also used, except for the patients’ age and the numbers of surgically-treated levels that were analyzed using the nonpaired Welch’s t-test. A p value <0.05 was considered to be statistically significant.

## Results

Prevalence of pseudoarthrotic spondylolysis in patients with lumbar degenerative disease who underwent spinal surgery

The mean age of the 354 patients was 63.2 ± 14.4 years-old, and the male/female ratio was 199/155. The prevalence of lumbar pseudoarthrotic spondylolysis in the 354 patients was 6.50% (23/354) (Table 1). Of the 23 patients having spondylolysis, bilateral spondylolysis was found in 19 patients and unilateral spondylolysis was found in four patients. One of these patients had spondylolysis at two levels (L4 and L5) and the levels of spondylolysis were two at L4, and 22 at L5. Lytic spondylolisthesis was observed only at one level caudal to the spondylolysis in 12 patients.

**Table 1:** Age, sex, and percentage of spondylolysis in patients who underwent lumbar surgery (n=354). M = male, F = female.

Age	(years)	63.2 ± 14.4 (18-91)
Sex	(M/F)	199 / 155
Spondylolysis	(%)	6.50% (23/354)

Thirteen of the 23 patients received surgical treatment at one level caudal to the spondylolysis (nine lytic spondylolisthesis, three disc herniation, and one discogenic pain). The remaining 10 patients received surgical treatment on other lumbar levels, which may not have been related to the existence of pseudoarthrotic spondylolysis.

**Table 2:** Characteristics of patients in the spondylolysis group who underwent surgery at one level caudal to the spondylolysis (lysis). M = male, F = female, TLIF: transforaminal lumbar interbody fusion.

Patient No.	Sex	Age (yr)	Level of lysis	Laterality of lysis	Diagnosis	Vertebral Slip	Surgery
1	M	71	L5	bilateral	Lytic Spondylolisthesis	+	TLIF
2	M	35	L5	bilateral	Disc Herniation	-	TLIF
3	M	39	L5	bilateral	Disc Herniation	+	Herniotomy
4	M	58	L5	unilateral	Lytic Spondylolisthesis	+	TLIF
5	M	44	L5	bilateral	Discogenic Pain	-	TLIF
6	M	62	L5	bilateral	Lytic Spondylolisthesis	+	TLIF
7	M	58	L5	bilateral	Lytic Spondylolisthesis	+	TLIF
8	F	72	L5	bilateral	Lytic Spondylolisthesis	+	TLIF
9	M	56	L5	bilateral	Disc Herniation	-	Herniotomy
10	F	45	L5	bilateral	Lytic Spondylolisthesis	+	TLIF
11	M	48	L4	bilateral	Lytic Spondylolisthesis	+	TLIF
12	M	71	L5	bilateral	Lytic Spondylolisthesis	+	TLIF
13	F	63	L5	bilateral	Lytic Spondylolisthesis	+	TLIF

Males showed significantly higher prevalence of spondylolysis (9.5%, 19/199 patients) than females (2.6%, 4/155 patients). The prevalence of spondylolysis in each disease state was as follows: lumbar disc herniation (8.9%, 10/112 patients); spondylolisthesis (6.8%, 9/133 patients); spinal stenosis (2.0%, 2/101 patients); and others (25%, 2/8 patients). Patients with lumbar disc herniation (p=0.058) and spondylolisthesis (p=0.087) showed a non-significant tendency toward higher prevalence of spondylolysis compared to patients with spinal stenosis.

Comparison between patients with pseudoarthrotic spondylolysis and without spondylolysis

**Figure 2:**
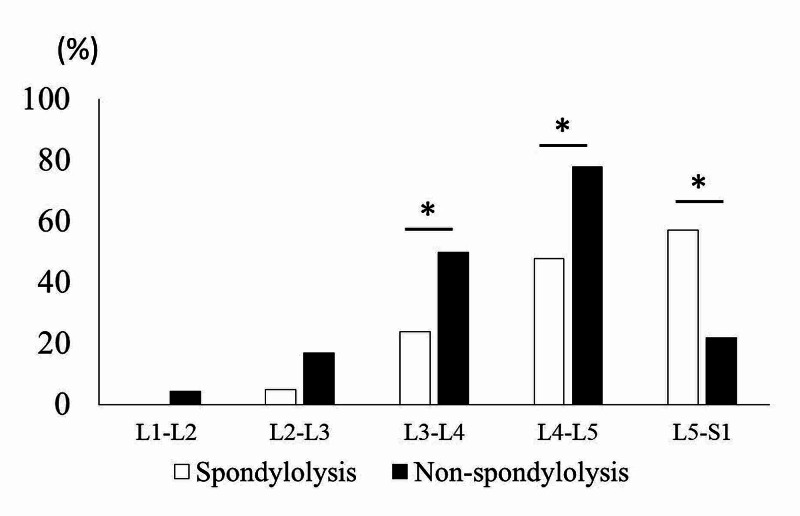
The percentages of patients who received surgery at each lumbar level. Patients with L5 spondylolysis (Spondylolysis) more frequently underwent spinal surgery at the L5-S1 level, but less frequently underwent spinal surgery at the L3-L4 and L4-L5 levels, when compared with patients without spondylolysis (Non-spondylolysis). Asterisks indicate statistical significance (p<0.05).

Of the 23 patients with pseudoarthrotic spondylolysis, 21 patients who had spondylolysis only at the L5 level were assigned to the spondylolysis group. Patients without spondylolysis were assigned to the non-spondylolysis group (n=331). When comparing the two groups, the patients’ age was significantly lower in the spondylolysis group (spondylolysis: 54.2 ± 13.5 years-old, non-spondylolysis: 63.8 ± 14.2 years-old), and the male/female ratio was significantly higher in the spondylolysis group (spondylolysis: 17/4, non-spondylolysis: 180/151) (Table 3). The number of surgically treated levels was significantly lower in the spondylolysis group (spondylolysis: 1.33 ± 0.56 levels, non-spondylolysis: 1.70 ± 0.87 levels) (Table 3). Although no significant difference was found (p=0.078), the percentage of patients who underwent multilevel spinal surgery was lower in the spondylolysis group (28.6%, 6/21 patients) than in the non-spondylolysis group (48.3%, 160/331 patients); this suggests that patients with pseudoarthrotic spondylolysis less frequently receive multilevel lumbar surgery (Table 3). The details of the surgically treated levels are shown in Figure 2. As shown in Figure 2, the percentage of patients who underwent surgery at L5-S1 was significantly higher in the spondylolysis group, whereas the percentage of patients who underwent surgery at L3-L4 or L4-L5 was significantly higher in the non-spondylolysis group. These results suggest that patients tend to undergo spinal surgery at the L5-S1 level more frequently when they have spondylolysis at the L5 level. On the other hand, patients tend to undergo spinal surgery at the L3-L4 and L4-L5 levels less frequently when they have spondylolysis at the L5 level.

**Table 3:** Characteristics of patients in the spondylolysis group and the non-spondylolysis group. (M/F): male/female

	Spondylolysis Group (n=21)	Non-spondylolysis Group (n=331)	p value
Age (years)	54.2 ± 13.5	63.8 ± 14.2	p < 0.01
Sex (M/F)	17/4	180/151	p < 0.05
Number of surgically-treated levels	1.33 ± 0.56	1.70 ± 0.87	p < 0.05
Single-level surgery/ multilevel surgery	15/6	171/160	P=0.078

## Discussion

The prevalence of pseudoarthrotic spondylolysis in the Japanese general population had been reported to be 5.9%, and males (7.9%) showed a higher prevalence than females (3.9%) [9]. We also recently studied the prevalence of spondylolysis in the general population using CT data from patients undergoing CT scans of abdominal or lumbar regions for reasons other than low back disorders, and reported similar results to the previous report (total: 6.4%, male: 7.7%, female: 4.5%) [11].

In the present study, lumbar degenerative disease patients requiring spinal surgery showed similar prevalence of spondylolysis (6.5%) compared to the general population (5.9-6.4%) [9,11]. Therefore, these data suggest that the occurrence of severe degenerative lumbar disease requiring spinal surgery in patients with pseudoarthrotic spondylolysis is not higher than that in patients without spondylolysis. These results suggest that the rate of patients who develop lumbar spinal disorders requiring future surgery may not increase when bony union is not achieved in acute spondylolysis patients. In patients with lumbar degenerative disease requiring spinal surgery, the difference in the prevalence of spondylolysis between males (9.5%) and females (2.6%) was more evident than that in the general population. Further study is needed to clarify whether the influence of spondylolysis on the prevalence of lumbar degenerative disease is different between males and females. Our study is the first to describe whether pseudoarthrotic spondylolysis increases the risk for developing severe spinal disorders requiring future spinal surgery.

The results of the present study indicate that patients tend to undergo surgery at an earlier age when patients have pseudoarthrotic spondylolysis. We had previously reported that patients with spondylolysis develop spondylolisthesis more often and earlier than patients without spondylolysis [11]. When limited to patients over 60 years old, the prevalence of spondylolisthesis was extremely high (90%, 18/20) in bilateral spondylolysis patients, indicating that bilateral spondylolysis is a strong risk factor for future occurrence of spondylolisthesis [11]. The presence of spondylolysis may accelerate lumbar degenerative change at the caudal disc at a younger age. Our results showed that patients with L5 spondylolysis more frequently develop severe degenerative disease requiring spinal surgery at the L5-S1 level; however, they less frequently develop such disease at L3-L4 and L4-L5 levels. Because mechanical load to lumbar intervertebral disc increases [12], spondylolysis patients have higher future incidences of disc degeneration and spondylolisthesis at one level caudal to the spondylolysis [6-11]. On the other hand, our results suggest that the presence of L5 spondylolysis reduces the risk for developing degenerative disease at a level other than the L5-S1 level. Sairyo et al. reported that the presence of pseudoarthrotic spondylolysis reduces mechanical stress to the adjacent cranial segment, which may be one of the reasons why L5 spondylolysis reduces the risk of adjacent segment degeneration (L3-L4, and L4-L5) [13]. More importantly, our results also suggest that the presence of spondylolysis may reduce the risk of multilevel lumbar disease requiring spinal surgery.

This study has several limitations. First, the study design was cross-sectional and not prospective. However, it takes a very long time to get results from prospective studies, because spondylolysis usually occurs in patients younger than 20 years old [14-16]. We, therefore, should suggest an estimated influence of pseudoarthrotic spondylolysis from the results of cross-sectional or retrospective studies. Second, the conclusion of the present study was based on the comparison of the data with previous studies showing the prevalence of spondylolysis in the general population [9,11]. However, one of the previous studies used for comparison is a study from our institution. Thus, the patients' data was collected in the same medical zone, and we could confirm that the patients' demographic data were similar to those of the present study [11]. Although there are several limitations, the results of our study provide information regarding the influence of pseudoarthrotic spondylolysis on the development of lumbar degenerative disease. Such information is useful for both physicians and patients when considering the treatment of acute spondylolysis.

## Conclusions

Pseudoarthrotic spondylolysis may not be significantly correlated with the occurrence of degenerative lumbar spinal disorders requiring spinal surgery; however, more patients tend to undergo spinal surgery at a younger age when they have pseudoarthrotic spondylolysis. The presence of pseudoarthrotic spondylolysis increases the risk for developing severe lumbar degenerative disease at one level caudal to the spondylolysis, but, it may reduce the risk at other levels. Interestingly, the presence of pseudoarthrotic spondylolysis may reduce the risk for developing multilevel lumbar degenerative disease requiring spinal surgery.

In conclusion, spondylolysis is not a risk factor for developing lumbar spinal disorders requiring future spinal surgery. When we treat patients with acute spondylolysis, the first aim of treatment is fracture healing. However, when patients choose not to stop sports activity or conservative treatment fails, sufficient information about suggested prognosis should be provided to avoid anxiety.
